# Inferring phenotypic causal structure among farrowing and weaning traits in pigs

**DOI:** 10.1111/asj.13369

**Published:** 2020-04-22

**Authors:** Toshihiro Okamura, Kazuo Ishii, Motohide Nishio, Guilherme J. M. Rosa, Masahiro Satoh, Osamu Sasaki

**Affiliations:** ^1^ Institute of Livestock and Grassland Science NARO Tsukuba Ibaraki Japan; ^2^ Department of Animal Sciences University of Wisconsin‐Madison Madison WI USA; ^3^ Department of Biostatistics and Medical Informatics University of Wisconsin‐Madison Madison WI USA; ^4^ Graduate School of Agricultural Sciences Tohoku University Aoba Sendai Japan

**Keywords:** inductive causation, phenotypic causal structure, pig, reproduction, structural equation model

## Abstract

Direct selection for litter size or weight at weaning in pigs is often hindered by external interventions such as cross‐fostering. The objective of this study was to infer the causal structure among phenotypes of reproductive traits in pigs to enable subsequent direct selection for these traits. Examined traits included: number born alive (NBA), litter size on day 21 (LS21), and litter weight on day 21 (LW21). The study included 6,240 litters from 1,673 Landrace dams and 5,393 litters from 1,484 Large White dams. The inductive causation (IC) algorithm was used to infer the causal structure, which was then fitted to a structural equation model (SEM) to estimate causal coefficients and genetic parameters. Based on the IC algorithm and temporal and biological information, the causal structure among traits was identified as: NBA → LS21 → LW21 and NBA → LW21. Owing to the causal effect of NBA on LS21 and LW21, the genetic, permanent environmental, and residual variances of LS21 and LW21were much lower in the SEM than in the multiple‐trait model for both breeds. Given the strong effect of NBA on LS21 and LW21, the SEM and causal information might assist with selective breeding for LS21 and LW21 when cross‐fostering occurs.

## 1 INTRODUCTION

In the swine industry, the number of weaning piglets and their total weight are particularly important traits. Direct selection for these traits is often restricted in practice due to external interventions such as cross‐fostering, whereby piglets are transferred between sows to equalize litter size. As a result, the number of nursing piglets at a sow can be different from the number of farrowing piglets. The purpose of cross‐fostering is to reduce piglet mortality at preweaning (Straw, Dewey, & Burgi, 1998). This management technique, however, can make it difficult to adequately estimate genetic parameters for litter size and weight at weaning (Su, Lund, & Sorensen, 2007). Therefore, in actual pig breeding, weaning is improved by selecting for total number born (Sorensen, Vernersen, & Andersen, 2000), number born alive (NBA) (Holl & Robinson, 2003), and litter size on day 5 based on the biological dam (Nielsen, Su, Lund, & Madsen, 2013; Su et al., 2007). These traits are not or only slightly affected by cross‐fostering because they are generally evaluated before or just after it. However, the genetic correlation between total number born and mortality remains unfavorable, and selection for higher total number born will increase the number of stillborn piglets. Putz, Tiezzi, Maltecca, Gray, and Knauer (2015) suggested that a higher amount of cross‐fostering reduced the genetic correlation between litter sizes in different days after farrowing. In the absence of cross‐fostering, selection could rely directly on the number of weaning piglets and their total weight. However, the beneficial effect of cross‐fostering on piglets’ survival increases selection intensity and makes selection more efficient because more candidates of a specific sire and dam can be obtained.

Gianola and Sorensen (2004) adapted the structural equation model (SEM) to mixed‐effects models in quantitative genetics to convey causal relationships among traits. Valente, Rosa, Gianola, Wu, and Weigel (2013) suggested that SEM made it possible to predict the effects of external interventions. Importantly, uncovering this information among reproductive traits in pigs might be useful for adequately evaluating weaning traits even in the case of cross‐fostering. For such evaluation the causal structure among preweaning and weaning traits should be determined first, but previous studies about causal structure among reproductive traits in pig have not focused on weaning traits (Chitakasempornkul et al., 2019; Varona, Sorensen, & Thompson, 2007).

The objective of this study was to infer the causal structure and estimate causal coefficients among phenotypes of farrowing and weaning traits in two breeds of pigs. This study is the first to describe a phenotypic causal structure among farrowing and weaning reproductive traits in pigs.

## 2 MATERIALS AND METHODS

### 2.1 Ethical statement

Approval of Animal Care and Use Committee was not required for this study because the data were acquired from an existing database.

### 2.2 Data

Data from Landrace and Large White populations from two farms for the years 2001–2017 were provided by CIMCO Corporation. All matings were performed by artificial insemination. Landrace, Large White, and Duroc sire breeds were used for matings. The NBA was recorded 1 day after farrowing and it took into account the number of piglets that seemed to be alive at farrowing but were actually dead. Records suspected of cross‐fostering or with missing values were not included in the analyses. Overall, the final dataset comprised of 6,240 litters from 1,673 Landrace dams and 5,393 litters from 1,484 Large White dams. Parity varied from one to eight. Pedigree data for Landrace and Large White sows included reproductive data on 2,102 dams and 1,849 sires.

Reproductive traits were NBA, litter size on day 21 (LS21), and litter weight on day 21 (LW21) after farrowing. Descriptive statistics for each trait are presented in Table 1.

**TABLE 1:** Descriptive statistics of reproductive traits

Trait	Landrace			Large White		
	NBA	LS21	LW21	NBA	LS21	LW21
*N*	6,240	6,240	6,240	5,393	5,393	5,393
Mean	10.81	9.25	52.49	10.40	9.15	53.33
*SD*	2.90	2.47	12.30	2.71	2.39	12.94
Minimum	1.00	1.00	4.00	1.00	1.00	4.60
Maximum	20.00	17.00	93.20	20.00	16.00	88.70

Abbreviations: LS21, litter size on day 21; LW21, litter weight on day 21; NBA, number born alive.

### 2.3 Statistical analyses

A multiple‐trait animal model (MTM) was used for initial analysis. The following MTM was considered:

y=Xβ+Zu+Wc+e,

where ***y*** is a vector of observations; ***β*** is a vector of systematic effects, including farrowing year (17 levels), farrowing month (12 levels), parity (8 levels), farm (2 levels), and mating sire breed (3 levels); ***u*** is a vector of random additive genetic effects; ***c*** is a vector of permanent environmental effects on the dams; ***e*** is a vector of random residuals; and ***X***, ***Z***, and ***W*** are known incidence matrices.

The joint distribution of the random vectors ***u***, ***c***, and ***e*** was given by:

$$\left(\begin{array}{c} u\\ c\\ e \end{array}\right)=N\left[\left(\begin{array}{c} 0\\ 0\\ 0 \end{array}\right),\left(\begin{array}{ccc} G_{0}⊗A & 0 & 0\\ 0 & C_{0}⊗I & 0\\ 0 & 0 & R_{0}⊗I \end{array}\right)\right],$$

where ***G*_0_** is the additive genetic (co)variance matrix, ***A*** is the additive (numerator) genetic relationship matrix, ***C*_0_** is the permanent environmental (co)variance matrix, ***I*** is an identity matrix with suitable dimensions, and ***R*_0_** is the residual (co)variance matrix. Such (co)variance matrices can be expressed as:

$$G_{0}=\left(\begin{array}{ccc} \sigma_{u_{1}}^{2} & \sigma_{u_{1}u_{2}} & \sigma_{u_{1}u_{3}}\\ \sigma_{u_{1}u_{2}} & \sigma_{u_{2}}^{2} & \sigma_{u_{2}u_{3}}\\ \sigma_{u_{1}u_{3}} & \sigma_{u_{2}u_{3}} & \sigma_{u_{3}}^{2} \end{array}\right),\;C_{0}=\left(\begin{array}{ccc} \sigma_{c_{1}}^{2} & \sigma_{c_{1}c_{2}} & \sigma_{c_{1}c_{3}}\\ \sigma_{c_{1}c_{2}} & \sigma_{c_{2}}^{2} & \sigma_{c_{2}c_{3}}\\ \sigma_{c_{1}c_{3}} & \sigma_{c_{2}c_{3}} & \sigma_{c_{3}}^{2} \end{array}\right),\;R_{0}=\left(\begin{array}{ccc} \sigma_{e_{1}}^{2} & \sigma_{e_{1}e_{2}} & \sigma_{e_{1}e_{3}}\\ \sigma_{e_{1}e_{2}} & \sigma_{e_{2}}^{2} & \sigma_{e_{2}e_{3}}\\ \sigma_{e_{1}e_{3}} & \sigma_{e_{2}e_{3}} & \sigma_{e_{3}}^{2} \end{array}\right)$$

where $\sigma_{u_{i}}^{2}$ is the additive genetic variance of trait *i*, $\sigma_{u_{i}u_{j}}$ is the additive genetic covariance between traits *i* and *j*, $\sigma_{c_{i}}^{2}$ is the permanent environmental variance of trait *i*, $\sigma_{c_{i}c_{j}}$ is the permanent environmental covariance between traits *i* and *j*, $\sigma_{e_{i}}^{2}$ is the residual variance of trait *i*, $\sigma_{e_{i}e_{j}}$ is the residual covariance between traits *i* and *j*, and *i* and *j* for 1, 2, and 3 represent NBA, LS21, and LW21, respectively.

The program GIBBS2F90 (Misztal et al., 2002) was used to fit the model within a Bayesian approach and to implement Gibbs sampling in order to obtain samples from posterior distributions of genetic, permanent environmental, and residual (co)variances. A total of 1,000,000 samples were generated; however, 500,000 were discarded as a conservative burn‐in and the remaining samples were thinned every 10 iteration, resulting in a total of 50,000 samples for posterior analysis. The results of Geweke's diagnostic (Geweke, 1992) and effective sample size derived by the program POSTGIBBSF90 (Misztal et al., 2002) were used to assess convergence with their recommended criteria.

### 2.4 The inductive causation algorithm

The inductive causation (IC) algorithm was applied to the residual (co)variances obtained from MTM analysis to infer a potential causal structure among the three traits, as already proposed by Valente, Rosa, de los Campos, Gianola, and Silva (2010). The residual (co)variances obtained from the MTM provide information from the joint distribution of phenotypic traits conditional on genetic effects. This method corrects for confounding effects when traits are genetically correlated (Rosa et al., 2011; Valente et al., 2010). The IC algorithm performs a series of statistical decisions based on partial correlations (*ρ*) between traits and consists of the following three steps (Pearl, 2000):

Step 1: Based on *ρ*, a statistical decision is made as to whether two traits are connected by an undirected edge. If *ρ* conditioning of the combination of all other traits between traits Y1 and Y2 differs from 0, Y1 and Y2 are connected by an undirected edge (Y1–Y2).

Step 2: Based on *ρ*, a statistical decision is made about the existence of an unshielded collider. When three traits Y1, Y2, and Y3 are connected with undirected edges to form a trio such as Y1–Y2–Y3, whereby two nonadjacent traits (Y1 and Y3) have a common adjacent trait (Y2), and Y1 and Y3 are conditionally dependent on any possible set that includes the adjacent trait Y2, the edges should be oriented toward the common adjacent trait; for example, Y1 → Y2 ← Y3, then Y2 is considered an unshielded collider. If Y1 depends on Y3 or Y3 depends on Y1 (i.e., Y3 → Y1 or Y1 → Y3) in this case, Y2 is considered a shielded collider, which the IC algorithm cannot detect because Y1 and Y3 are always conditionally dependent on any possible set.

Step 3: When possible, the remaining undirected edges are oriented so that no new unshielded colliders or cycles are introduced.

Statistical decisions regarding whether to declare *ρ* as null or not were based on from 75% to 95% highest posterior density (HPD) intervals, in 5% increments. If the intervals contained the value 0, the correlation was declared null. The analysis was performed using an R (R Development Core Team, 2009) script written by Valente and Rosa (2013).

### 2.5 SEM analysis

The SEM was fitted to the causal network inferred by the IC algorithm. The model can be described as:

$$y=\Lambda ⊗Iy+{X\beta }^{*}+{\mathrm{Zu}}^{*}+{\mathrm{Wc}}^{*}+e^{*},$$

with the joint distribution of vectors u^*^, c^*^, and e^*^ as:

$$\left(\begin{array}{c} u^{*}\\ c^{*}\\ e^{*} \end{array}\right)=N\left[\left(\begin{array}{c} 0\\ 0\\ 0 \end{array}\right),\left(\begin{array}{ccc} G_{0}^{*}⊗A & 0 & 0\\ 0 & \Gamma_{0}^{*}⊗I & 0\\ 0 & 0 & \Psi_{0}^{*}⊗I \end{array}\right)\right]$$

where vectors ***y***, β^*^, u^*^, c^*^, e^*^, ***X***, ***Z***, ***A***, and ***I*** have a similar meaning as described above for the MTM. However, here, these vectors represent systematic and random effects directly affecting each trait, that is, effects that are not mediated by other phenotypic traits (Gianola & Sorensen, 2004; Rosa et al., 2011; Valente et al., 2013). Additionally, ***Λ*** is a 3 × 3 matrix with 0 on the diagonal and with structural coefficients (linear effects between pairs of traits) or 0 on the off‐diagonals, $G_{0}^{*}$ is the SEM additive genetic (co)variance matrix (it describes variance and covariances of direct genetic effects), $\Gamma_{0}^{*}$ is a diagonal matrix with the SEM permanent environmental variances, and $\Psi_{0}^{*}$ is a diagonal matrix with the SEM residual variances. These permanent environmental and residual covariances were assumed to be 0 in the SEM. Such direct SEM (co)variance matrices can be expressed as:

$$G_{0}^{*}=\left(\begin{array}{ccc} \sigma_{u_{1}^{*}}^{2} & \sigma_{u_{1}^{*}u_{2}^{*}} & \sigma_{u_{1}^{*}u_{3}^{*}}\\ \sigma_{u_{1}^{*}u_{2}^{*}} & \sigma_{u_{2}^{*}}^{2} & \sigma_{u_{2}^{*}u_{3}^{*}}\\ \sigma_{u_{1}^{*}u_{3}^{*}} & \sigma_{u_{2}^{*}u_{3}^{*}} & \sigma_{u_{3}^{*}}^{2} \end{array}\right),\;\Gamma_{0}^{*}=\left(\begin{array}{ccc} \sigma_{c_{1}^{*}}^{2} & 0 & 0\\ 0 & \sigma_{c_{2}^{*}}^{2} & 0\\ 0 & 0 & \sigma_{c_{3}^{*}}^{2} \end{array}\right),\;\Psi_{0}^{*}=\left(\begin{array}{ccc} \sigma_{e_{1}^{*}}^{2} & 0 & 0\\ \sigma_{e_{1}e_{2}} & \sigma_{e_{2}^{*}}^{2} & 0\\ 0 & 0 & \sigma_{e_{3}^{*}}^{2} \end{array}\right).$$

where $\sigma_{u_{i}^{*}}^{2}$ is the additive genetic variance of trait *i*, $\sigma_{u_{i}^{*}u_{j}^{*}}$ is the additive genetic covariance between traits *i* and *j*, $\sigma_{c_{i}^{*}}^{2}$ is the permanent environmental variance of trait *i*, $\sigma_{e_{i}^{*}}^{2}$ is the residual variance of trait *i*, and *i* and *j* for 1, 2, and 3 represent NBA, LS21, and LW21, respectively.

This model was fitted to estimate genetic parameters and causal coefficients representing causal effects with the assumed causal structure mentioned above. The covariances of permanent environmental and residual effect were conducted as diagonal to achieve parameter identifiability. Importantly, in the SEM, the causal parents of a given trait (e.g., Y1 is the causal parent of Y2 in Y1 → Y2) are included as covariates in the SEM assigned to that trait. The program GIBBS2F90 was used to fit this model and to obtain posterior samples for SEM parameters; a Gibbs sampling strategy similar to that used for the MTM was employed here as well.

## 3 RESULTS

The posterior means and 95% HPD intervals describing ***G*_0_**, ***C*_0_**, and ***R*_0_** obtained with the MTM for each breed are listed in Table 2. No major differences were observed between Landrace and Large White pigs in terms of genetic and permanent environmental (co)variances. However, the residual variance of NBA was larger in Landrace than in Large White pigs with no 95% HPD interval overlap observed between breeds. In contrast, residual variance of LW21 and covariance between LS21 and LW21 were greater in Large White than Landrace.

**TABLE 2:** Posterior means and 95% highest posterior density (HPD) intervals for the dispersion parameters pertaining to the multiple‐trait animal model

Parameter	Landrace			Large White		
	Posterior mean	95% HPD interval		Posterior mean	95% HPD interval	
		Low	High		Low	High
$\sigma_{u_{1}}^{2}$	0.98	0.60	1.38	0.87	0.48	1.28
$\sigma_{u_{1}u_{2}}$	0.66	0.38	0.97	0.62	0.30	0.94
$\sigma_{u_{1}u_{3}}$	1.26	−0.01	2.53	1.69	0.09	3.33
$\sigma_{u_{2}}^{2}$	0.63	0.37	0.89	0.57	0.29	0.84
$\sigma_{u_{2}u_{3}}$	1.74	0.66	2.85	2.37	0.96	3.84
$\sigma_{u_{3}}^{2}$	19.90	13.39	26.81	29.76	19.51	40.19
$\sigma_{c_{1}}^{2}$	0.50	0.23	0.79	0.68	0.39	1.00
$\sigma_{c_{1}c_{2}}$	0.42	0.19	0.64	0.54	0.30	0.79
$\sigma_{c_{1}c_{3}}$	1.62	0.63	2.57	2.16	0.92	3.36
$\sigma_{c_{2}}^{2}$	0.40	0.20	0.61	0.45	0.24	0.67
$\sigma_{c_{2}c_{3}}$	2.01	1.16	2.91	2.12	1.02	3.19
$\sigma_{c_{3}}^{2}$	14.59	9.42	19.77	17.24	9.97	24.54
$\sigma_{e_{1}}^{2}$	5.85	5.62	6.09	4.92	4.70	5.14
$\sigma_{e_{1}e_{2}}$	4.06	3.88	4.25	3.70	3.51	3.88
$\sigma_{e_{1}e_{3}}$	9.67	8.98	10.36	10.53	9.75	11.35
$\sigma_{e_{2}}^{2}$	4.59	4.40	4.77	4.37	4.18	4.56
$\sigma_{e_{2}e_{3}}$	13.30	12.63	13.98	15.25	14.39	16.06
$\sigma_{e_{3}}^{2}$	83.54	80.14	86.96	107.28	102.40	112.00

Note

$\sigma_{c_{i}}^{2}$, permanent environmental variance of trait *i*; $\sigma_{c_{i}c_{j}}$, permanent environmental covariance between traits *i* and *j*; $\sigma_{e_{i}}^{2}$, residual variance of trait *i*; $\sigma_{e_{i}e_{j}}$, residual covariance between traits *i* and *j;* $\sigma_{u_{i}}^{2}$, additive genetic variance of trait *I*; $\sigma_{u_{i}u_{j}}$, additive genetic covariance between traits *i* and *j*, and *i* and *j* for 1, 2, and 3 represent number born alive, litter size on day 21, and litter weight on day 21, respectively.

**FIGURE 1:**
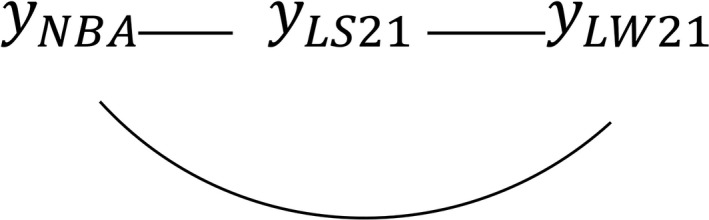
Undirected graph detected by the inductive causation algorithm with 95% HPD intervals. LS21, litter size on day 21; LW21, litter weight on day 21; NBA, number born alive

**FIGURE 2:**
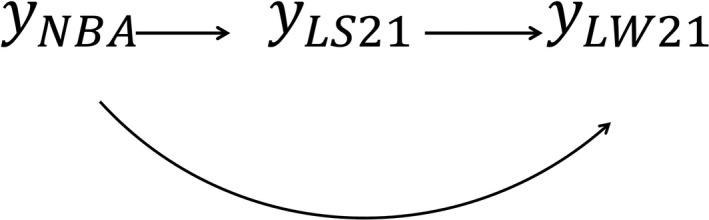
Directed graph assumed based on Figure 1 plus temporal and biological information. LS21, litter size on day 21; LW21, litter weight on day 21; NBA, number born alive

Based on the posterior distribution of ***R*_0_**, *ρ* between traits was estimated and applied in Step 1 for causal structure search using different HPD interval contents. None of the HPD intervals contained 0, indicating that the three traits were connected by an undirected edge (Figure 1). In our case, there was no unshielded collider because none of the traits were “unshielded”. Therefore, Step 2 of the IC algorithm was not able to indicate any link. This suggested the IC algorithm could not detect any directed edges among these traits. Then, temporal prior knowledge about the relationship between NBA and LS21 or LW21 was applied to specify directions, that is, NBA → LS21 and NBA → LW21, as NBA is expressed before LS21 or LW21. We also applied prior biological knowledge about the relationship between LS21 and LW21, that is, LS21 → LW21, meaning that litter size affects total litter weight because the vice versa is generally difficult to be explained by a biological system. Hence, we assumed the causal structure shown in Figure 2, which describes the additive effect of genetic, permanent environmental, and residual parameters.

The dispersion parameters and their structural coefficients were fitted into the SEM with the assumed structure (Figure 2) and are presented in Tables 3 and 4. A comparison of dispersion parameters for each trait between MTM (Table 2) and SEM (Table 3) revealed that all variances for LS21 and LW21 (but not NBA) in the SEM were lower than in the MTM. These differences were found in both breeds although some residual variances differed significantly between them. Here, *λ_i,j_* denotes a structural coefficient from the *j*th to the *i*th trait; this means that when the *j*th trait increases by 1 unit, the *i*th trait increases by *λ_i,j_* units. The direct effect of NBA on LS21 (*λ*_LS21, NBA_) was positive. The indirect effect of NBA on LW21 via LS21 (*λ*_LS21, NBA_ × *λ*_LW21, LS21_) was also positive, but the direct effect of NBA on LW21 (*λ*_LW21, NBA_) was negative. Finally, the total effect of NBA on LW21 (*λ*_LW21, NBA_ + *λ*_LS21, NBA_ × *λ*_LW21, LS21_) was positive. Although all coefficients were significantly different between both breeds, their signs were always in the same direction.

**TABLE 3:** Posterior means and 95% highest posterior density (HPD) intervals for the dispersion parameters pertaining to the structural equation model

Parameter	Landrace			Large White		
	Posterior mean	95% HPD interval		Posterior mean	95% HPD interval	
		Low	High		Low	High
$\sigma_{u_{1}^{*}}^{2}$	0.93	0.55	1.34	0.82	0.43	1.21
$\sigma_{u_{1}^{*}u_{2}^{*}}$	0.03	−0.07	0.12	−0.02	−0.11	0.07
$\sigma_{u_{1}^{*}u_{3}^{*}}$	0.26	−0.39	0.91	0.56	−0.21	1.37
$\sigma_{u_{2}^{*}}^{2}$	0.20	0.13	0.27	0.13	0.08	0.18
$\sigma_{u_{2}^{*}u_{3}^{*}}$	0.66	0.35	0.97	0.80	0.46	1.13
$\sigma_{u_{3}^{*}}^{2}$	14.54	10.72	18.45	18.44	13.17	23.85
$\sigma_{c_{1}^{*}}^{2}$	0.53	0.23	0.82	0.71	0.42	1.02
$\sigma_{c_{2}^{*}}^{2}$	0.07	0.02	0.12	0.06	0.02	0.10
$\sigma_{c_{3}^{*}}^{2}$	5.65	3.11	8.11	7.65	4.28	11.09
$\sigma_{e_{1}^{*}}^{2}$	5.85	5.62	6.09	4.92	4.70	5.13
$\sigma_{e_{2}^{*}}^{2}$	1.76	1.69	1.83	1.58	1.51	1.64
$\sigma_{e_{3}^{*}}^{2}$	42.99	41.21	44.69	50.98	48.73	53.19

Note

$\sigma_{c_{i}^{*}}^{2}$, permanent environmental variance of trait *i*; $\sigma_{e_{i}^{*}}^{2}$, residual variance of trait *I*; $\sigma_{u_{i}^{*}}^{2}$, additive genetic variance of trait *I*; $\sigma_{u_{i}^{*}u_{j}^{*}}$ , additive genetic covariance between traits *i* and *j*, and *i* and *j* for 1, 2, and 3 represent number born alive, litter size on day 21, and litter weight on day 21, respectively.

**TABLE 4:** Posterior means and 95% highest posterior density (HPD) intervals for the causal coefficients pertaining to the structural equation model

Parameter	Landrace			Large White		
	Posterior mean	95% HPD interval		Posterior mean	95% HPD interval	
		Low	High		Low	High
λ_LS21,NBA_	0.70	0.68	0.71	0.75	0.74	0.77
λ_LW21,NBA_	−0.96	−1.08	−0.84	−1.33	−1.49	−1.17
λ_LW21,LS21_	3.78	3.65	3.92	4.65	4.48	4.82
λ_LW21,LS21_ × λ_LS21,NBA_	2.64	2.53	2.75	3.50	3.34	3.65
λ_LW21,NBA_ + λ_LW21,LS21_ × λ_LS21,NBA_	1.68	1.58	1.77	2.16	2.04	2.29

Note

λ*_i,j_*, structural coefficient of *j* on *i*; LS21, litter size on day 21; LW21, litter weight on day 21; NBA, number born alive.

## 4 DISCUSSION

The objective of this study was to investigate the causal structure and estimate the causal coefficients among preweaning and weaning traits in pigs, which could then be used for direct selection based on litter size and litter weight at weaning when cross‐fostering is employed. Although it was impossible to artificially change the empirical NBA values, we assumed that this artificial change could be done by cross‐fostering just after farrowing and NBA assessment. This assumption allowed us to determine the causal structure among NBA, LS21, and LW21, as well as estimate the causal coefficients.

The IC algorithm was based on 95% HPD intervals and returned a completely undirected structure. This result indicated that the structure was very reliable because the level for statistical decision was high and all causal coefficients were still statistically different from 0. Although many studies have reported a genetic relationship among reproductive traits (Chen, Baas, Mabry, Koehler, & Dekkers, 2003; Putz et al., 2015; Roehe & Kennedy, 1995), only a few have described phenotypic causal structures among reproductive traits in pigs. For example, Varona et al. (2007) observed a negative causal relationship between litter size and average piglet weight at birth in Large White animals. Chitakasempornkul et al. (2019) used the IC algorithm to infer a causal phenotypic network among reproductive traits at birth and found a negative relationship between NBA and average piglet body weight, with the latter negatively affecting the total number born during subsequent gestation in gilts. However, they did not focus on weaning traits and this study is the first to describe a phenotypic causal structure among farrowing and weaning reproductive traits in pigs. In addition, the structure described here was common in both Landrace and Large White breeds, suggesting that it may be stable among different breeds in spite of differences in magnitude of causal coefficients. In fact, these differences indicate that such coefficients may need to be estimated for each population or breed.

In our structure, NBA could directly affect LS21 and LW21, and indirectly affect LW21 via LS21. This means that LS21 was not only affected directly by $u_{\text{LS21}}^{*}$ but also indirectly by $u_{\text{NBA}}^{*}$. LW21, on the other hand, was affected directly by $u_{\text{LW21}}^{*}$ and indirectly by both $u_{\text{NBA}}^{*}$ and $u_{\text{LS21}}^{*}$. We believe that $u_{\text{NBA}}^{*}$ might represent genes affecting uterine size or ovulation rate, $u_{\text{LS21}}^{*}$ may include genetic effects determining the piglets’ survival (e.g., quality and quantity of colostrum or piglet crushing), and $u_{\text{LW21}}^{*}$ may represent total volume and quality of milk produced (Valente et al., 2013). Cross‐fostering can affect the estimation of genetic effects associated with these traits in the MTM because direct effects cannot be distinguished and can vary with the number of nursing piglets. If the number of nursing piglets is artificially changed by cross‐fostering, these direct genetic effects can be accurately estimated by the SEM because in this case direct genetic effects are independent of the number of nursing piglets. Therefore, inferring a causal structure can benefit direct selection based on LS21 and LW21 when cross‐fostering is applied.

The genetic, permanent environmental, and residual variances of LS21 and LW21 in the SEM were smaller than in the MTM in both breeds because components of these effects were different in the two models. In the MTM, the effects represent overall effects, which include all direct and indirect (i.e., mediated by other phenotypic traits) effects on each trait. In contrast, the effects represent only direct effects in the SEM (i.e., not mediated by other traits in the causal structure) (Valente et al., 2013). The reduction in variance of downstream traits conditioning upstream traits was in agreement with the results observed for bovine milk fatty acid (Bouwman, Valente, Janss, Bovenhuis, & Rosa, 2014) and bovine meat quality (Inoue et al., 2016). The substantial reduction in variances of LS21 and LW21 indicated that these two traits were strongly affected by NBA. Accordingly, an artificial change (i.e., an external intervention) in the number of nursing piglets by cross‐fostering could strongly affect the phenotype and estimated breeding values of LS21 and LW21. In addition, its usefulness in breeding for LS21 and LW21 with cross‐fostering on computer simulation or real data should be confirmed in the future.

In general, residual covariance matrix is constructed as diagonal to achieve parameter identifiability. The permanent environmental and residual covariance matrices were set as diagonal in the SEM of this study. Although this restriction was strong it was necessary to identify the parameters, previous studies used the same method to identify them (Bouwman et al., 2014; Inoue et al., 2016; Rosa et al., 2011; Valente et al., 2010). However, when the covariances among permanent environmental effects or among residual effects would not be 0, the causal effects would be over‐ or underestimated. Such covariances can be taken account by the statistical model with proper fixed and random effects, but we cannot find whether the model was sufficient. Therefore, it is noted that the parameter could have resulted in bias in this study.

In conclusion, we describe here the causal structure among NBA, LS21, and LW21 based on the IC algorithm and temporal and biological information. The causal structure allowed for the estimation of causal coefficients. A comparison of dispersion parameters for LS21 and LW21 between the MTM and SEM indicated that the phenotype arising from LS21 and LW21 traits could be strongly affected by the NBA trait. This finding suggests that cross‐fostering could have a big impact on LS21 and LW21, and thus the causal information might be useful for direct selection based on LS21 and LW21 when cross‐fostering is employed.
